# Comparison of electrical impedance tomography and spirometry-based measures of airflow in healthy adult horses

**DOI:** 10.3389/fphys.2023.1164646

**Published:** 2023-07-05

**Authors:** David P. Byrne, Ben Keeshan, Giselle Hosgood, Andy Adler, Martina Mosing

**Affiliations:** ^1^ School of Veterinary Medicine, Murdoch University, Perth, WA, Australia; ^2^ Department of Systems and Computer Engineering, Carleton University, Ottawa, ON, Canada; ^3^ Anaesthesiology and Perioperative Intensive Care, Department for Companion Animals and Horses, University of Veterinary Medicine, Vienna, Austria

**Keywords:** asthma, equine, expiratory, inspiratory, peak flow, plethysmography

## Abstract

Electrical impedance tomography (EIT) is a non-invasive diagnostic tool for evaluating lung function. The objective of this study was to compare respiratory flow variables calculated from thoracic EIT measurements with corresponding spirometry variables. Ten healthy research horses were sedated and instrumented with spirometry via facemask and a single-plane EIT electrode belt around the thorax. Horses were exposed to sequentially increasing volumes of apparatus dead space between 1,000 and 8,500 mL, in 5–7 steps, to induce carbon dioxide rebreathing, until clinical hyperpnea or a tidal volume of 150% baseline was reached. A 2-min stabilization period followed by 2 minutes of data collection occurred at each timepoint. Peak inspiratory and expiratory flow, inspiratory and expiratory time, and expiratory nadir flow, defined as the lowest expiratory flow between the deceleration of flow of the first passive phase of expiration and the acceleration of flow of the second active phase of expiration were evaluated with EIT and spirometry. Breathing pattern was assessed based on the total impedance curve. Bland-Altman analysis was used to evaluate the agreement where perfect agreement was indicated by a ratio of EIT:spirometry of 1.0. The mean ratio (bias; expressed as a percentage difference from perfect agreement) and the 95% confidence interval of the bias are reported. There was good agreement between EIT-derived and spirometry-derived peak inspiratory [−15% (−46–32)] and expiratory [10% (−32–20)] flows and inspiratory [−6% (−25–18)] and expiratory [5% (−9–20)] times. Agreement for nadir flows was poor [−22% (−87–369)]. Sedated horses intermittently exhibited Cheyne-Stokes variant respiration, and a breath pattern with incomplete expiration in between breaths (*crown-like* breaths). Electrical impedance tomography can quantify airflow changes over increasing tidal volumes and changing breathing pattern when compared with spirometry in standing sedated horses.

## 1 Introduction

Determination of changes in airflow is important for understanding respiratory physiology and evaluating clinical airway disease such as asthma. Equine asthma is a significant cause of airflow limitation in affected horses, often leading to poor performance and occasionally more serious clinical signs such as respiratory distress (Bullone and Lavoie, 2020). Airflow in horses is reliably measured using spirometry (Nolen-Walston et al., 2009; Dixon et al., 2021). Spirometry in conscious horses requires the use of a facemask. Depending on the design of the mask, this may lead to rebreathing of expired gas and adverse reactions of the horse if they have not been accustomed to mask-wearing (Sides et al., 2018). Masks have also been shown to cause hypoxemia, hypercapnia, altered upper airway pressures and reduced respiratory rates in exercising horses (Evans and Rose, 1988; Holcombe et al., 1996).

Electrical impedance tomography (EIT) is a non-invasive functional imaging modality that has been used to describe changes in distribution of ventilation in horses (Ambrisko et al., 2016; Mosing et al., 2016; Ambrisko et al., 2017; Mosing et al., 2017; Mosing et al., 2018; Auer et al., 2019; Crivellari et al., 2021; Sacks et al., 2021; Brabant et al., 2022). It has been used with increasing frequency in the assessment of ventilated humans with COVID-19 (Tomasino et al., 2020; Chen et al., 2021) as well as in humans with acute respiratory distress syndrome (Scaramuzzo et al., 2020), particularly to titrate positive end-expiratory pressure and thus optimize ventilation. It has also been shown to detect changes in airflow in horses undergoing histamine-induced bronchoprovocation and subsequent salbutamol-induced bronchodilation as well as exercise induced changes (Secombe et al., 2020; Herteman et al., 2021; Secombe et al., 2021). Electrical impedance tomography can thus assess flow non-invasively in a number of settings and species (Brabant et al., 2022). Beside the benefit of not requiring a facemask and horses being used to having a belt around the thorax, a key advantage of EIT over spirometry is its ability to provide regional data. Emerging evidence in both humans and horses has demonstrated differences in airflow between regions of the lungs in health and disease (Zhao et al., 2012; Zhao et al., 2013; Frerichs et al., 2016; Secombe et al., 2020; 2021).

There are limited data comparing flow variables derived from EIT and spirometry. A previous study in children showed strong correlation between global EIT and spirometry-derived flow-volume loops (Ngo et al., 2018). A second study in 7 healthy adult humans showed strong correlation and agreement during spontaneous breathing and forced expiration maneuvers for forced expiratory volume in one second (FEV_1_) and FEV_1_/functional residual capacity (Ngo et al., 2017). A secondary analysis of intubated mechanically-ventilated adult humans demonstrated good correlation between peak inspiratory and expiratory flows measured by EIT and spirometry (Mauri et al., 2019). Similar findings in mechanically-ventilated healthy pigs and pigs with experimentally-induced lung injury identified good correlation and clinically-relevant agreement (Bodenstein et al., 2014).

The aim of the study was to evaluate the agreement between EIT and spirometry derived airflow variables in healthy adult sedated horses over a variety of tidal volumes. The hypothesis was that airflow variables derived from EIT can be extrapolated to spirometry-based values with clinically-acceptable accuracy (defined as the 95% confidence interval of the variation in agreement generally not exceeding 25% above or below perfect agreement).

## 2 Materials and methods

This study was approved by the Animal Ethics Committee of Murdoch University (permit number R3258/20) and performed according to the Animal Welfare Act of Western Australia (2003). All animals were cared for according the Australian code for care and use of animals for scientific purposes (https://www.nhmrc.gov.au/about-us/publications/australian-code-care-and-use-animals-scientific-purposes, 2013; Accessed 23 May, 2022).

As this was an exploratory observational study, our sample size was based on the size of the variance that could be tolerated to derive estimates considered valuable. A sample size of 10 would allow estimation of 95% confidence intervals within +/- 25% of the mean, given a variance of up to 40% of the mean. These horses (8 Standardbreds and 2 Thoroughbreds) were from the institutional teaching herd and were identified as clinically healthy based on history and a thorough physical examination with special focus on the respiratory tract before inclusion into the study group. The horses included 3 mares and 7 geldings, with a mean (SD) age of 13.1 (3.6) years and body weight of 500 (49) kg. The median (range) body condition score was 5.5/9 (4-7/9) (Henneke et al., 1983). Horses were chosen based on temperament. If horses objected strenuously to instrumentation despite sedation, they were to be removed from the study.

Each horse was walked into stocks from the paddock to which they were accustomed through regular habituation therein. An 18-gauge 4 cm intravenous catheter (Surflo, Terumo, Philippines) was placed aseptically in a jugular vein. The horse was sedated with xylazine (Ilium Xyazil-100, Troy Laboratories, Glendenning, NSW, Australia) (0.5–1 mg/kg) IV and sedation maintained with a constant rate infusion (CRI) of xylazine (0.69 mg/kg/h) (Ringer et al., 2012). The hair coat behind the elbow, at the level of the 6^th^ intercostal space at mid thorax was circumferentially wettened with water to improve electrode contact. A custom-made neoprene single-plane EIT belt with 32 equidistantly mounted electrodes was placed around the chest at this location for the duration of the procedure. A small amount of non-conductive ultrasound gel was placed on each electrode prior to placement. The belt was connected to the EIT device (BBvet, SenTec AG, Landquart, Switzerland) which applied a skip-4 method of current injection to the electrodes. EIT data were displayed and recorded using the accompanying software (BBvet, SenTec AG, Landquart, Switzerland). A face mask (Aeromask, Trudell Medical International, Ontario, Canada) was placed on the horse’s muzzle and a Fleisch no. 5 pneuomotachograph connected to a commercial equine spirometry system (OpenPleth; Ambulatory Monitoring Inc, Ardsley, NY, United States) was attached to a customized port on the face mask to measure airflow. Spirometry data was recorded electronically using the included software system (Equine Flowmetrics, Ambulatory Monitoring Inc, Ardsley, NY, United States). The spirometer was calibrated with a 3L calibration syringe (Model 5630 series, Hans Rudolph Inc., Shawnee, KS, United States) prior to each horse. A side-stream capnograph (Surgivet V9203 multiparameter monitor, Sound Veterinary Equipment, Australia) was attached to the face mask via a customized port.

Baseline (T_BL_) EIT and spirometry data were collected prior to the addition of a series of tubing of increasing volume to the distal end of the spirometer, to induce rebreathing of CO_2_ and thus an increase in ventilation. The purpose of this rebreathing apparatus was to enable assessment of agreement of airflow across a clinically-relevant range of tidal volumes. This rebreathing apparatus was increased in size at each timepoint to provide the following equipment dead space volumes: 1,000 mL (T_1000_), 2000 mL (T_2000_), 3,500 mL (T_3500_), 6,000 mL (T_6000_), 8,500 mL (T_8500_). For all timepoints, EIT and spirometry data were collected after at least 2 min of stabilization (defined by a stable inspired CO2 concentration ± 2 mmHg), and at least 10 stable breaths, or 2 min, was recorded. Inspired and expired CO_2_, heart rate, respiratory rate and tidal volume calculated from the spirometer were recorded at the midpoint of each recording. Additional boluses of 0.25 mg/kg xylazine were administered as required.

Once the tidal volume exceeded 150% of the baseline, or clinical hyperpnea (defined as an increase in respiratory efforts identifiable as obvious use of respiratory accessory muscles including flaring of nostrils with prominent thoracic retraction) was appreciated, the rebreathing apparatus was removed at the end of that data collection period, and the horse allowed to recover. Additional data were collected during this recovery period (T_REC_) where all of the additional apparatus dead space was removed but the mask kept in place, four to 5 minutes after removal of the dead space. After the horses reached their baseline RR and end-tidal CO2 concentration the xylazine CRI was stopped. Horses were then de-instrumented and allowed to fully recover from sedation before returned to the paddock.

### 2.1 Data analysis

#### 2.1.1 Synchronization of EIT and spirometry data

From the global impedance signal, which has a linear relationship with inhaled gas volume, the first derivative was calculated as inspiratory and expiratory airway flow (F_EIT_). Previously generated algorithms were used to calculate global airway flow over time (Secombe et al., 2020; Secombe et al., 2021). The waveform of the measured flow from spirometry (F_SPIRO_) and F_EIT_ were evaluated from the beginning of the synchronously begun recordings. No further time corrections were applied. The F_SPIRO_ waveform was corrected for baseline drift over time. The spirometry volume signal was calculated by integrating the F_SPIRO_ signal and detrending the resulting curve from temperature-related error over time. The fEIT flow signal was calculated from the EIT volume curve as follows: first, the signal was scaled with a calibration factor to best fit the fEIT (in arbitrary units, AU) to the volume curve derived from the spirometry data; next, a Savisky-Golay filter was used to calculate a smoothed time derivative to represent flow. This filter is a common way to calculate derivatives of noisy data without introducing phase delays. It should be noted that this scaling was only performed for the first breath, and that these breaths were only selected for analysis on a small number of occasions, thus minimizing the effects of scaling one measurement to the other. Thus, no further correction of spirometry and EIT waveforms was applied after the first breath. Where periods of apnea separated selected breaths (and thus segmented the data), scaling was performed for each segment and recorded.

#### 2.1.2 Breath selection

Breath selection from EIT ventilation data was performed via visual evaluation of the global impedance curve using IbeX software (Ibex, Sentec, CH-4106 Therwil BL, Switzerland). For each recording, Custom software was written in MATLAB (Mathworks, Natick, MA, United States) to perform the filtering and calibration steps outlined above. The software then calculated flow and timing variables for each breath. Next, ten breaths with a regular breath waveform were manually selected in EIT and spirometry data and analysed in MATLAB.

#### 2.1.3 Flow signal calculation

**FIGURE 1:**
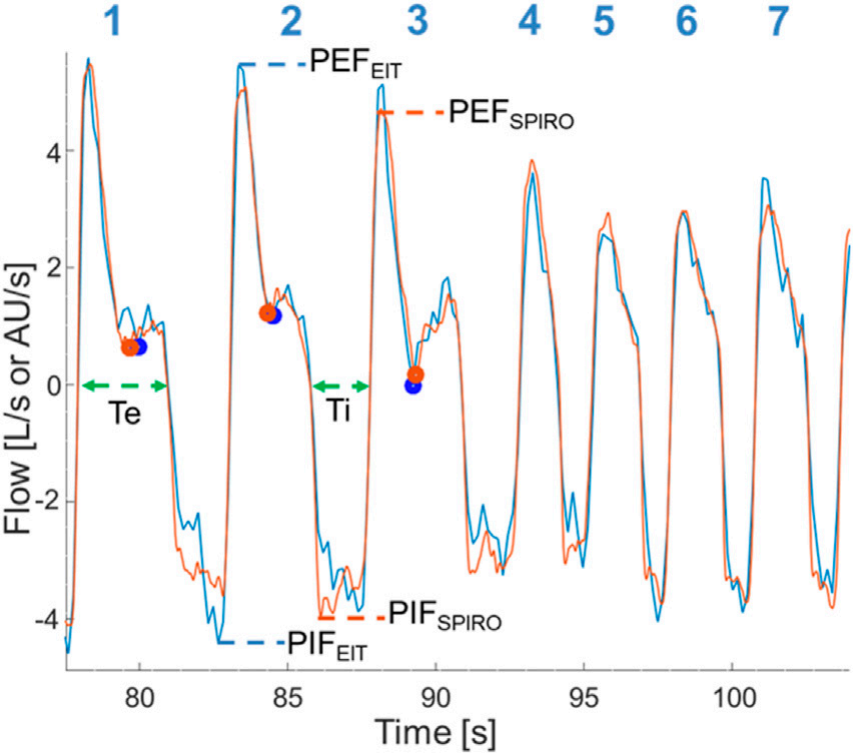
Superimposition of EIT flow curve (blue) and spirometry curve (orange) after time synchronizing, correction of baseline spirometry drift, and rescaling of the EIT flow signal. Data is from horse 6 at T_2000_. Breath numbers are above each corresponding flow curve. Dots denote the expiratory nadir flow (blue: EIT-derived; orange: spirometry-derived). Dashed lines indicate flow-derived data (blue: EIT-derived; orange: spirometry-derived; green: data from both EIT and spirometry). Breaths 4-7 have no clear expiratory nadir flow and a faster respiratory rate is present. Ti, Inspiratory time; Te, Expiratory time; PIF_SPIRO_/_EIT_, Peak inspiratory flow derived from spirometry or EIT, respectively; PEF_SPIRO_/_EIT_, Peak expiratory flow derived from spirometry or EIT, respectively.

Peak inspiratory (PIF) and expiratory flows (PEF), inspiratory (Ti) and expiratory times (Te), were calculated from both datasets. For EIT and spirometry, PIF and PEF were calculated at the maximum inflection points of the inspiratory and expiratory flow curves. For EIT, Ti was measured from the start of the negative (inspiratory) impedance derivative change to the end of the negative impedance derivate change and Te was measured from the start of the positive (expiratory) impedance derivative change to the end of the positive (expiratory) impedance derivative change. For spirometry, Ti was measured from the beginning of negative flow below baseline to the return to baseline and Te defined as the time from this point until the crossing of the baseline (as some expiratory flows briefly reached baseline). A novel variable called expiratory nadir flow was evaluated and defined as the lowest expiratory flow between the deceleration of flow of the first passive phase of expiration and the acceleration of flow of the second active phase of expiration, or, if no acceleration was present in the second phase of expiration, the intercept of the first and second phase (Figure 1).

Breathing patterns and breath morphology in the global impedance waveform were evaluated in the recorded segments at each time point for each horse. Visual analysis of the global impedance curves revealed an abnormal breathing pattern comprising gradually changing cycles of large impedance breaths to small impedance breaths with or without periods of apnea. The impedance curve occasionally demonstrated atypical breath morphology where the end-expiratory lung impedance (EELI) between breaths remained higher than the EELI from the first and final breath of the cluster. The occurrence of these breathing patterns and breath morphology was recorded.

#### 2.1.4 Statistics

Means for all variables from all breaths at each timepoint were calculated and used for analyses. Data was assessed for normality using the Shapiro-Wilkes test and visual inspection of Q-Q plots. Normal data is reported as mean ± SD, or mean [95% Confidence Interval (CI)] and non-normal data as median (range). Where breath selection occurred across multiple segments within a timepoint (and thus had multiple calibrations) that timepoint was excluded. Where breath selection by the algorithm was inaccurate for an individual breath, as visually determined from the EIT and spirometry volume or flow curves, respectively, all variables for both modalities for that breath only, were excluded from analysis. The EIT and spirometry variables were evaluated for agreement using Bland Altman analysis for multiple measurements per subject with the ratio of EIT variables to spirometry variables plotted against the spirometry measurement for each variable, except expiratory nadir flow. The mean ratio (bias), 95% CI of the bias and the 95% CI of these limits were calculated. For expiratory nadir flow only, the difference between the EIT measurement and the spirometry measurement was plotted against the spirometry measurement with the mean difference (bias) and the 95% confidence interval of the bias calculated. This was to account for times when the nadir flow was at zero, or briefly negative (i.e., inspiratory) litres per second. Results are expressed as a percentage difference from perfect agreement. Medcalc (Medcalc Software Ltd., 8,400 Ostend Belgium) was used for all analyses.

## 3 Results

### 3.1 General results

All horses completed the experiment, and none was excluded due to temperament. All horses completed T_BL_–T_3500_ before reaching one of the two predefined end points. Five and 3 horses completed T_6000_ and T_8500_, respectively. The following horse-timepoints were deleted due to breath selection failure occurring across multiple segments with multiple calibrations: T_BL_–horse 1 and 8; T_1000_—horse 1; T_2000_—horse 1 and 8; T_3500_—horse 8. Data were missing for one horse for T_REC_ (horse 7). The expiratory nadir flow algorithm failed to detect a change in the flow slope during expiration at two timepoints in two horses, despite one being present on visual flow curve examination (horse 6 and 9 for T_8500_ and T_REC_, respectively). Scale correction factors for the EIT flow signal are reported in Supplementary Table S1.

Standard parameters are reported in Table 1. The duration of the experiment was 74 ± 13 min. The total xylazine dose was 1.67 ± 0.24 mg/kg. Two horses required additional boluses of 0.25 mg/kg and one horse required two additional boluses each of 0.125 mg/kg.

**TABLE 1:** Standard parameters for horses at each timepoint (T_BL_, Baseline; T_1000–8500_, 1,000–8,500 mL additional apparatus dead space; T_REC_, recovery after removal of additional apparatus dead space).

Timepoint	HR (bpm)	RR (bpm)	VT_spiro_ (l)	InCO_2_ (mmHg)	etCO_2_ (mmHg)
T_BL_ (*n* = 8)	29 ± 3	7 (5–17)	7.38 ± 1.69	1.7 ± 1.4	42.9 ± 9.1
T_1000_ (*n* = 9)	29 ± 2	7 (6–12)	7.59 ± 1.72	2.8 ± 2.9	48.9 ± 4.1
T_2000_ (*n*-8)	30 ± 4	7.5 (6–12)	8.69 ± 1.9	3.6 ± 3.3	52.2 ± 2.1
T_3500_ (*n* = 9)	30 ± 3	7 (6–12)	9.42 ± 2.2	4.5 ± 4.1	52.4 ± 2.3
T_6000_ (*n* = 5)	30 ± 3	7.5 (6.5–9)	9.47 ± 1.51	5.6 ± 5.2	53.7 ± 3.1
T_8500_ (*n* = 3)	31 ± 4	7.75 (6–8)	9.81 ± 1.01	2.8 ± 1.9	54.1 ± 3.2
T_REC_ (*n* = 8)	31 ± 4	6 (4–9)	6.73 ± 1.29	1.6 ± 0.8	48.3 ± 3.1

HR, heart rate in beats per minutes (bpm); RR, respiratory rate in breath per minute (bpm); VT_spiro_, tidal volume as calculated by spirometry; InCO_2_, inspired carbon dioxide partial pressure; etCO_2_, end-tidal carbon dioxide partial pressure. Parameters are reported as mean ± SD, or median (range) according to data distribution.

### 3.2 Agreement F_SPIRO_ and F_EIT_ variables

**FIGURE 2:**
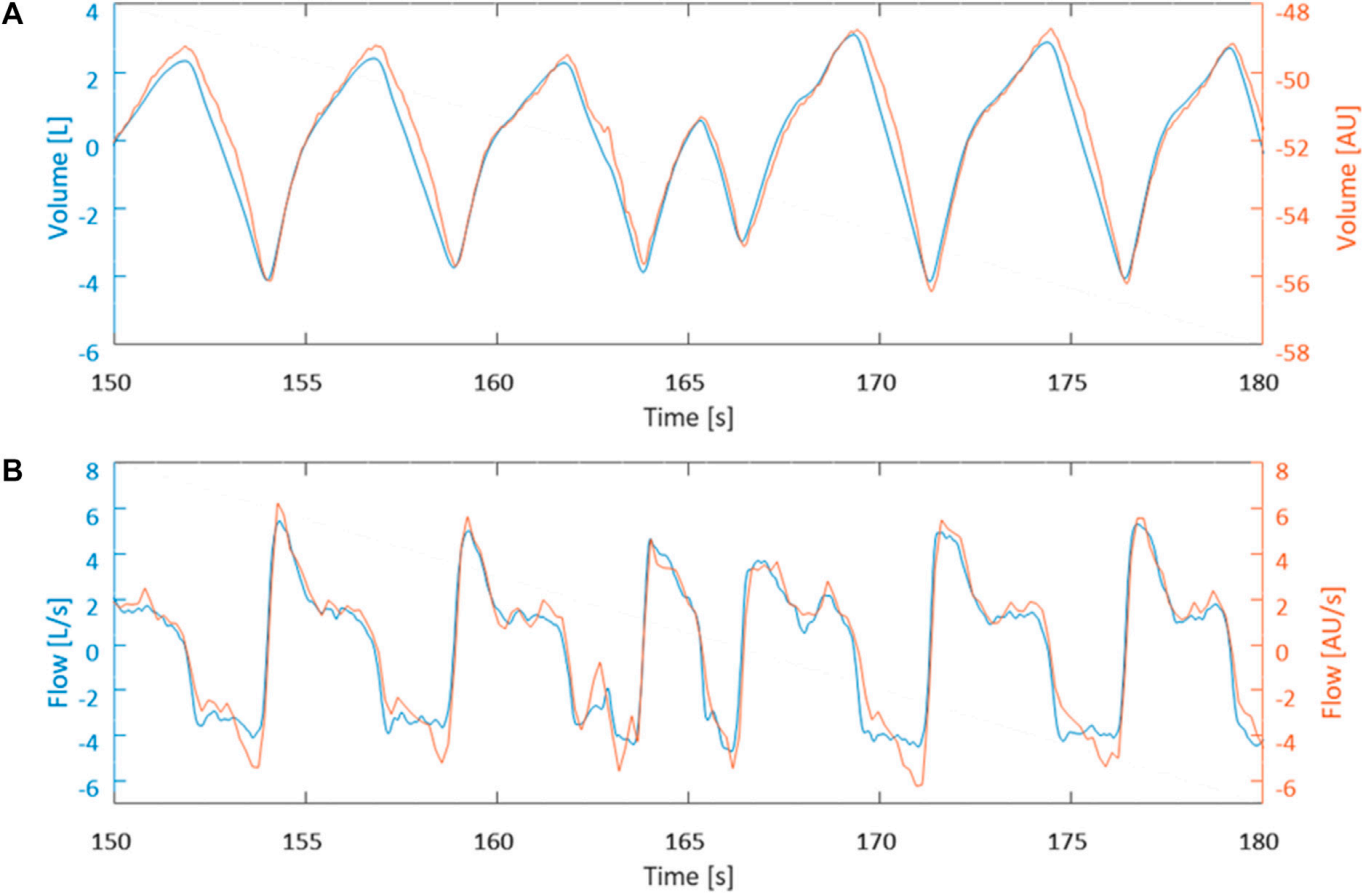
Data is from horse 1 at timepoint T_3500_. **(A)** Visual comparison between measured EIT volume curve (blue) and calculated spirometry volume curve (orange) after synchronization. **(B)** Visual comparison between calculated EIT flow curve (blue) and measured spirometry curve (orange) synchronization.

**FIGURE 3:**
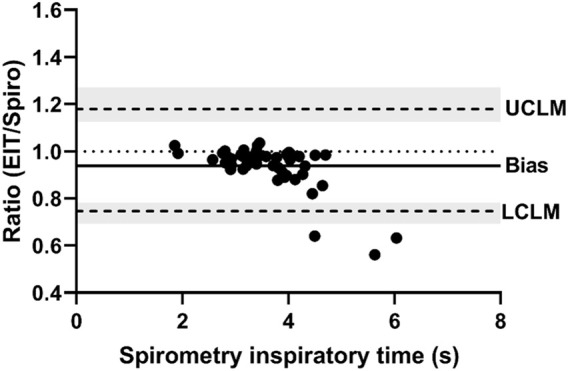
Bland-Altman plot of the ratio of EIT peak inspiratory flow (AU/s) to spirometry peak inspiratory flow (L/s) on the Y-axis and spirometry on the X-axis. The line of agreement is at 1 (dotted line). The mean EIT to spirometry ratio (bias) is 0.8471 (solid line) with a lower confidence limit (LCLM) of 0.544 (95% CI 0.4038–0.6315) and an upper confidence limit (UCLM) of 1.3191 (95% CI 1.1363–1.7768) indicated by hashed lines and shading.

**FIGURE 4:**
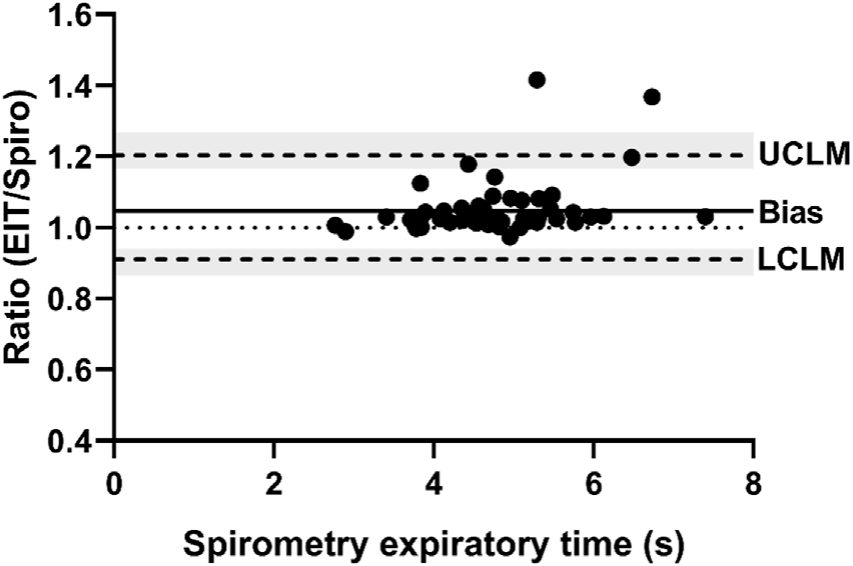
Bland-Altman plot of the ratio of EIT peak expiratory flow (AU/s) to spirometry peak expiratory flow (L/s) on the Y-axis and spirometry on the X-axis. The line of agreement is at 1 (dotted line). The mean EIT: spirometry ratio (bias) is 0.9039 (solid line) with a lower confidence limit (LCLM) of 0.6807 (95% CI 0.569–0.7452) and an upper confidence limit (UCLM) of 1.2003 (95% CI 1.0964–1.436) indicated by hashed lines and shading.

**FIGURE 5:**
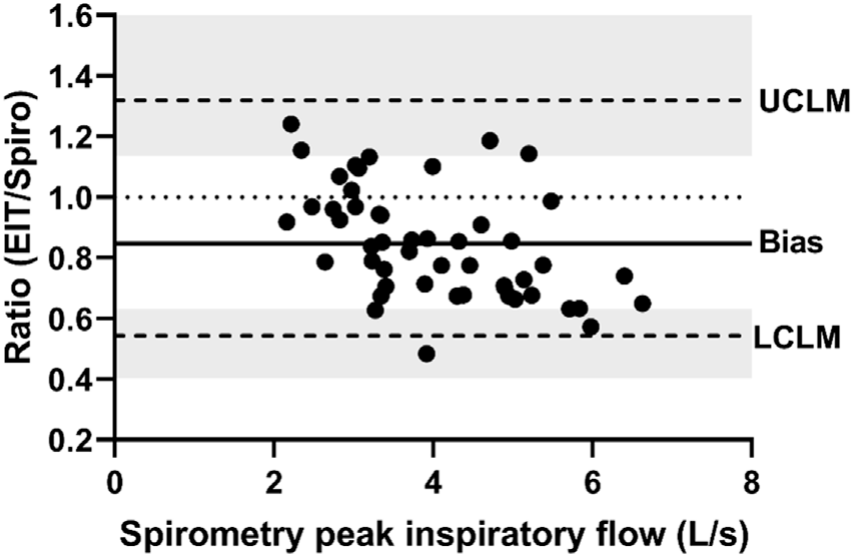
Bland-Altman plot of the ratio of EIT inspiratory time (s) to spirometry inspiratory time (s) on the Y-axis and spirometry inspiratory time on the X-axis. The line of agreement is at 1 (dotted line). The mean EIT: spirometry ratio (bias) is 0.9385 (solid line) with a lower confidence limit (LCLM) of 0.7465 (95% CI 0.6929–0.7824) and an upper confidence limit (UCLM) of 1.1799 (95% CI 1.1259–1.2711) indicated by hashed lines and shading.

**FIGURE 6:**
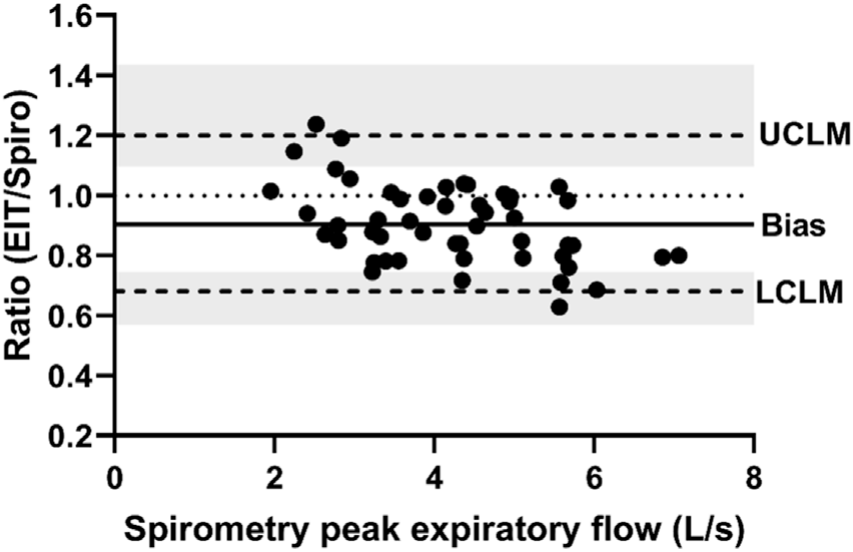
Bland-Altman plot of the ratio of EIT expiratory time (s) to spirometry expiratory time (s) on the Y-axis and spirometry expiratory time on the X-axis. The line of agreement is at 1 (dotted line). The mean EIT: spirometry ratio (bias) is 1.0467 (solid line) with a lower confidence limit (LCLM) of 0.9105 (95% CI 0.8643–0.9392) and an upper confidence limit (UCLM) of 1.2033 (95% CI 1.1665–1.2676) indicated by hashed lines and shading.

**FIGURE 7:**
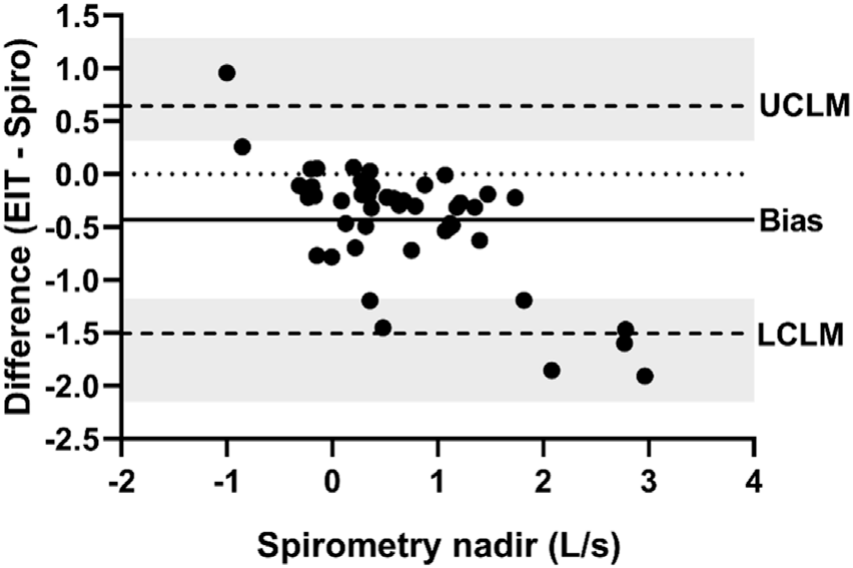
Bland-Altman plot of the ratio of EIT expiratory nadir flow (AU/s) to spirometry expiratory nadir flow (L/s) on the Y-axis and spirometry on the X-axis. The line of agreement is at 1 (dotted line). The mean EIT: spirometry ratio (bias) is 0.7763 (solid line) with a lower confidence limit (LCLM) of 0.1284 (95% CI 0.06307–0.1944) and an upper confidence limit (UCLM) of 4.6921 (95% CI 3.1006–9.5542) indicated by hashed lines and shading.

Figures 1, 2 demonstrate examples of the superimposed EIT and spirometry flow curves. Bland-Altman plots are presented in Figures 3–7. For PIF, the bias was −15.29% (95% CI −45.6–31.91). For PEF, the bias was −9.61% (95% CI −31.93–20.03). For Ti, the bias was −6.15% (95% CI −25.35–17.99). For Te, the bias was 4.67% (95% CI −8.95–20.33). For the expiratory nadir flow, the bias was −22.37% (95% CI −87.16–369.21).

### 3.3 Breathing pattern

**FIGURE 8:**
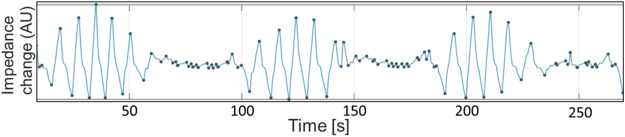
Global impedance curve from horse 10 during baseline breathing (T_BL_), demonstrating periods of apnea interspersed with breaths of gradually increasing and decreasing sizes. The small oscillations during apnea periods are cardiac-related signals. Impedance change in arbitrary units is displayed on the X-axis. Seconds are displayed on the Y-axis.

Sedation in 4/10 horses was associated with a breathing pattern consisting of decrescendo and crescendo amplitudes of tidal volumes (Naughton, 1998). In three horses, periods of apnea were observed in between the decrescendo and crescendo (Cheyne-Stokes respiration; Figure 8) while in one horse, small breaths were recorded in between a crescendo and decrescendo (Cheyne-Stoke variation). This breathing pattern was abolished by carbon dioxide rebreathing (three horses by T_2000_ and one horse by T_3500_). Two horses that exhibited this pattern at T_BL_ also exhibited this pattern upon recovery T_REC_.

**FIGURE 9:**
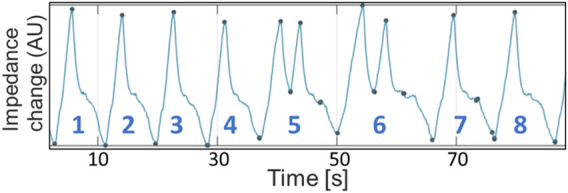
Global impedance curve from horse 2 during T_1000_ (initial rebreathing) demonstrating incomplete expiration prior to subsequent re-inspiration (breaths 5 and 6). Impedance change in arbitrary units is displayed on the X-axis. Seconds are displayed on the Y-axis.

Seven out of 10 horses exhibited intermittent single or multiple breaths with incomplete expiration in between each breath which were identified as *crown-like* breaths (Moreno-Martinez et al., 2022). One of the ten horses had *crown-like* breaths at T_BL_, five horses at T_1000_, two horses at T_2000_, T_3500_ and T_REC_, and two different individual horses at T_6000_ and T_8500_. The median (range) number of *crown-like* breaths per timepoint was 1 (1–4) (Figure 9).

## 4 Discussion

This study is the first that confirms the hypothesis that across a variety of tidal volumes, EIT-derived flow variables had clinically acceptable agreement with “gold-standard” spirometry-derived flow variables, with the 95% confidence interval of the variation in agreement generally not exceeding 25% above or below perfect agreement for inspiratory and expiratory times and peak flows. The novel variable, expiratory nadir flow, did not have clinically acceptable agreement between EIT and spirometry devices. Breathing pattern changed after sedation with Cheyne-Stokes respiration and *crown-like* breaths were observed.

Accurate airflow evaluation is an important variable in pulmonary function testing (PFT). In-field pulmonary function testing should be well tolerated, non-invasive, real-time and able to accurately detect disease. Most methods of pulmonary function testing in conscious horses currently require the use of facemasks. While the effects of a facemask on respiratory mechanics and breathing patterns at rest are unknown, there are effects at exercise (Evans and Rose, 1988; Holcombe et al., 1996). EIT has been used in people wearing high-flow oxygen cannulae (and thus unintubated) to assess changes in airflow when spirometry is not feasible (Mauri et al., 2017). Another limitation of existing pulmonary function testing methodologies when used in clinical cases is associated with tolerance of the mask, in comparison to a circumferential thoracic EIT electrode belt, to which most horses used for athleticism will be accustomed to wearing. One way to overcome the intolerance of a mask is by performing the PFT under sedation. Our study however shows that sedation can have a significant effect on breathing pattern and breath morphology and therefore does not support the use of sedatives for PFT. Furthermore, *crown-like* breaths have been observed in anaesthetized horses under triple drip anaesthesia and spirometry software was unable to evaluate > 20% of data sets with *crown-like* breaths which might bias the spirometry data during PFTs (Moreno-Martinez et al., 2022). At T_8500_, the inspired carbon dioxide concentration was unexpectedly low (mean 2.8 mmHg). However, only three horses were evaluated at this timepoint. It may be that end-tidal carbon dioxide is a better reflection of the actual effectiveness of rebreathing, which was highest at T_8500_.

Evidence in healthy and asthmatic children and healthy adults suggests that EIT and spirometry give comparable results for airway flow variables and forced expiratory maneuvers (Ngo et al., 2017; 2018). Global EIT-derived expiratory flow also correlates with spirometry data in people with obstructive lung disease, namely, cystic fibrosis (Zhao et al., 2012; Lehmann et al., 2016). While forced expiratory maneuvers have been described in horses (Couëtil et al., 2000), this test requires cooperation of the patient and therefore larger tidal volumes induced by rebreathing are typically used clinically for PFT in horses. We tried to simulate this situation by sequentially increasing rebreathing volume which resulted in an > 30% increase in tidal volume. The data in this study demonstrated that EIT can give similar flow measurements to spirometry, once calibrated, across a range of different tidal volumes, as a proxy for forced expiration maneuvers. In unintubated people, percentage changes of PIF and PEF have been calculated using EIT (Mauri et al., 2017), which negates the need for calibration. However, since it has been shown in multiple species and conditions that changes in the volume signal correlate with changes in tidal volume (Caruana et al., 2017; Ngo et al., 2017; Brabant et al., 2021; Crivellari et al., 2021; Mosing et al., 2022), calibration of EIT volume signal to calculated spirometry signal and subsequent derivation of flow signal would be expected to perform similarly to spirometry in assessing clinically relevant changes in airflow. Synchronisation of EIT and spirometry data was performed similarly to those in human evidence and translational medicine, save that volume data from mechanical ventilators were used to calibrate the EIT signal in those studies, rather than plethysmography (Bodenstein et al., 2014; Mauri et al., 2019).

Beside the routine PEF and PIF, a novel flow variable, the expiratory nadir flow, was described based on our observations of a distinct low flow section when the passive phase changes to the active phase of expiration on the spirometry and EIT curves (Figure 1; breaths 1–3). The expiratory nadir flow was chosen as EIT algorithms can detect changes in the slope of the flow curve (Secombe et al., 2021). While many timepoints visually demonstrated sufficient agreement in the expiratory nadir flow (Figure 8), there was no obvious change in slope during expiration in the spirometry curve in some horses at certain timepoints, which made a comparison impossible for these time points (Figure 1, breaths 4–7). The loss of this nadir in flow might be due to loss of one of the phases of expiration during sedation or rebreathing in some horses. As a comparison, horses with severe equine asthma have a loss of biphasic expiratory flow as the whole expiratory component of respiration becomes active (Hoffman et al., 2007). In our data, expiratory nadir flows seemed to occur less commonly with increased respiratory rates, another common occurrence in severe asthma. Expiratory nadir flow, despite having pathophysiologic relevance, may not be appropriate as a reliable marker of airway disease using current algorithms.

The bias of PEF and PIF was within 25% of perfect agreement, although the lower and upper confidence limits were not always. While the differences in observed measurements of PEF_EIT_ and PEF_SPIRO_ may be due entirely to error in measurement, another consideration is “real” physiologic differences due to the location of detection of airflow (see breaths 1-2, Figure 1). This error, where EIT underestimates peak flows as measured by spirometry, has been observed in humans and pigs who are intubated and mechanically-ventilated (Bodenstein et al., 2014; Mauri et al., 2019). Spirometry detects global flow at the nose, whereas EIT detects flow in a lens-shaped cross section of the thorax, typically at a section chosen to maximize the area of lung assessed (Frerichs et al., 2017; Brabant et al., 2022). Thus, there may be physiologic differences in the global and local airflow measured. Airway dead space has been shown to be a significant reason for differences between tidal volume measured by EIT and spirometry (Crivellari et al., 2021), and thus it would be expected that this would play a role on the volume-derived flow variables also. Furthermore, spirometry measures turbulent flow at the airway opening while flow calculations derived from EIT predominantly originate in region of the smaller airways where flow is mostly laminar and therefore peak flow might be different (West, 2012). More specifically, spirometry indirectly measures flow by evaluating the drop in pressure for a given glow resistance. Gas redistribution between different regions of the lung or gas trapping causing gas to stay undetected by the spirometer during expiration may also be responsible. Differences in peak flows between EIT and spirometry might also be due to cardiac related impedance signals falsifying the ventilatory impedance change (Ngo et al., 2018). However, this is not considered a likely cause due to the infrequent synchronization of cardiac-derived and lung impedance changes in horses. The small differences detected in our study between Ti and Te may relate to the differing frame rates between EIT and spirometry, where the EIT used in this study generates 47 frames a second while spirometry should have a sample rate of at least 100Hz (Miller et al., 2002). Interestingly, the same three horses were outliers for both Ti and Te, and so individual horse effects may also contribute.

Due to its ability to measure respiratory function over long periods of time, EIT is also able to detect physiologic phenomena that might be transient like altered breathing patterns, such as detected in this study. The abnormal breathing patterns during sedation (prior to or after rebreathing) are most consistent with Cheyne-Stokes (with apnea) or Cheyne-Stokes variant (without apnea) respiration, as the global impedance curves of each breath sequence between the apneic periods gradually increased and decreased. Cheyne-Stokes respiration is typically associated with heart failure or stroke in people due to increased sympathetic tone and is infrequently described in horses, typically after alpha-2 receptor agonists which can be classified as sympathomimetic drugs (Gleed and Dobson, 1988; Wagner et al., 2012). We gave xylazine hydrochloride, an alpha-2 receptor agonist, as a CRI sedative to facilitate rebreathing which likely explains the presence of Cheyne-Stokes respiration. We also observed altered breath morphology on the EIT impedance curve reported as *crown-like* breaths in anaesthetized horses (Kowalczyk et al., 2014; Moreno-Martinez et al., 2022). These breaths had an influence on the distribution of ventilation and shifted gas towards dependent parts of the lungs (Moreno-Martinez et al., 2022). Our study is the first to observe this phenomenon in conscious horses. However, changes in distribution of ventilation were not analysed as this was not the aim of this study.

Previous literature has demonstrated that EIT can detect changes in airflow, particularly during expiration, in horses with experimentally-induced bronchoconstriction (Secombe et al., 2020; Secombe et al., 2021) as well as asthmatic horses (Herteman et al., 2021). The results of this paper demonstrate that EIT can be used not only to evaluate relative changes in flow without calibration, but that EIT-derived calculated flow can also agree with flow measured by spirometry over a wide range of tidal volumes, when appropriately calibrated. Total impedance change has been shown to have a high linearity to tidal volume in horses, cattle and humans (Caruana et al., 2017; Ngo et al., 2017; Brabant et al., 2021; Crivellari et al., 2021; Mosing et al., 2022). Recently, EIT has been used to accurately estimate tidal volume after an indirect two-point calibration allowing the breath-by-breath evaluation of tidal volume with the EIT (Mosing et al., 2022). With this method of calibration, real-time flow expressed in L/sec (i.e., the same units as spirometry) may be feasible using EIT. Although not statistically demonstrable, the flow curves were visually more similar than the agreement between PIF and PEF suggests, indicating that EIT-calculated airflow may be useful even in the absence of calibration.

The main limitation relates to the retrospective application of the scaling factor applied to the flow waveform. This was necessary to scale the EIT flow signal back to the original volume signal size after derivation. However, given each scaling factor was individual for each timepoint (or multiple times within a timepoint), real-time flow determination requires further algorithmic development. Future studies should evaluate the magnitude of change in flow measured by EIT and spirometry over time rather than absolute values. This would allow verification of whether EIT can correctly detect alterations as it is unable to directly measure absolute values.

A limitation for translation of our findings to horses with airway disease is the inclusion of only healthy horses. However, the range of tidal volumes involved somewhat negates this limitation as high flows are observed in horses with higher clinical scores of asthma (Robinson et al., 2000) and thus rebreathing is likely to mimic flow rates seen in equine asthma. Furthermore, given the direct relationship between flow and volume, this is considered unlikely to significantly contribute to a lack of generalizability.

## 5 Conclusion

Electrical impedance tomography can be used to evaluate relative changes in airflow, and to estimate peak inspiratory and expiratory airflow when appropriately calibrated, in a non-invasive real-time manner in standing sedated horses. Derived times and peak flows from EIT are sufficiently accurate to potentially replace spirometry as a less invasive diagnostic tool, under certain circumstances.

## Data Availability

The raw data supporting the conclusion of this article will be made available by the authors, without undue reservation.
